# Prolegomena on the Neurosis, &c. &c.

**Published:** 1845-04

**Authors:** 


PART SECOND.

### Bibliographical Notices.

---

ART. I.—*Prolegomeni sulle Neurosi, &c. &c.* Memoria del Dottor Lorenzo Gemignani.—*Bologna*, 1843.

*Prolegomena on the Neuroses, &c. &c.* A Memoir by Dr. L. GEMIGNANI. *Read before the Medico-Chirurgical Society of Bologna.* (From Vol. iii, Fasc. iv, of the Trans. of the Society.)

THE Medico-Chirurgical Society of Bologna fixed one of the subjects for the Sgarze Prize Essay of 1842, as follows:

"To determine if neurosis be a morbid state essentially different from that common to all dynamic, irritative, and organico-chemical diseases of other structures of the system. In the essay must be laid down: 1, The most probable physiological doctrines of the general functions of the nervous system; 2, Whether nervous debility is of a specific nature, and originates in the nerves by the intervention of morbific causes, or whether the special phenomena which accompany them depend upon changes of certain particular modes of condition or action of the nerves; 3, Whether the so-called nervines act beneficially in the neuroses in virtue of a specific mode of action, or simply, as in other systems, by their elective action on the nervous systems."

The author of the essay before us was the only candidate for the prize; his labour was deemed worthy of "honorable mention" and of being printed.

Dr. Gemignani commences his essay by a general notice of the diverse and contradictory doctrines of modern neurologists. He enumerates no fewer than eight species: the tubular structure of the nervous system; the oscillations of the fibrils as electric conductors; the physiological independence of the nervous system in general, and of the ganglionic system specially; the reflex doctrines; the irritability of Glisson; excitability, &c.; and compares these one with another. Thus, for example, he observes that the doctrine of Pugginotti, that the ganglionic centres are the seat of the conservative instincts, is totally opposed to that of Müller and Hall; that the theory of the "tubulosity" of the nervous fibrils is incompatible with the theory of their being electric conductors, &c. The arguments for and against these views are developed in four successive chapters, with considerable ingenuity, but principally with reference to Italian authors and polemical controversies, for the most part unknown to us Tramontanes. Dr. Gemignani also intermingles a neat little theory of his own. There are a few novel views, as, for example, that the nerves are prolongations of the arteries, and the hypothetical inferences to be deduced therefrom. These, however, appear to us to be more ingenious than plausible; at least they are much too hypothetical to be admitted into our valuable pages. His general conclusions are, that the nervous fibrils are tubular (prolongations of the arterial trunks, as we have just now remarked), and contain "a peculiar fluid of a nature unknown, but probably the quintessence of the blood;" that primarily the nerves are formed thus, and the nervous system developed, from the periphery to the circumference; that the sensitive fibrils gradually form trunks as they pass to the cerebro-spinal axis; that arrived there, they enter into relations with the motor fibrils (the ultimate terminations of the *cerebral* arteries), and that reunited into nervous cords, they again pass from the brain into the spinal cord, and are thence distributed as motor nerves to all parts susceptible of motion.

Dr. Gemignani's pathology and therapeutics are altogether hypothetical, and without interest for the English reader.

